# Neighboring cells override 3D hydrogel matrix cues to drive human MSC quiescence

**DOI:** 10.1016/j.biomaterials.2018.05.032

**Published:** 2018-09

**Authors:** Silvia A. Ferreira, Peter A. Faull, Alexis J. Seymour, Tracy T.L. Yu, Sandra Loaiza, Holger W. Auner, Ambrosius P. Snijders, Eileen Gentleman

**Affiliations:** a Centre for Craniofacial and Regenerative Biology, King's College London, London SE1 9RT, UK; b Protein Analysis and Proteomics Platform, The Francis Crick Institute, London NW1 1AT, UK; c Cancer Cell Protein Metabolism Group, Department of Medicine, Imperial College London, London W12 0NN, UK

**Keywords:** Mesenchymal stem cell, Hydrogel, Quiescence, Extracellular matrix

## Abstract

Physical properties of modifiable hydrogels can be tuned to direct stem cell differentiation in a role akin to that played by the extracellular matrix in native stem cell niches. However, stem cells do not respond to matrix cues in isolation, but rather integrate soluble and non-soluble signals to balance quiescence, self-renewal and differentiation. Here, we encapsulated single cell suspensions of human mesenchymal stem cells (hMSC) in hyaluronic acid-based hydrogels at high and low densities to unravel the contributions of matrix- and non-matrix-mediated cues in directing stem cell response. We show that in high-density (HD) cultures, hMSC do not rely on hydrogel cues to guide their fate. Instead, they take on characteristics of quiescent cells and secrete a glycoprotein-rich pericellular matrix (PCM) in response to signaling from neighboring cells. Preventing quiescence precluded the formation of a glycoprotein-rich PCM and forced HD cultures to differentiate in response to hydrogel composition. Our observations may have important implications for tissue engineering as neighboring cells may act counter to matrix cues provided by scaffolds. Moreover, as stem cells are most regenerative if activated from a quiescent state, our results suggest that *ex vivo* native-like niches that incorporate signaling from neighboring cells may enable the production of clinically relevant, highly regenerative cells.

## 1 Introduction

Hydrogels formed from natural and synthetic polymers, which can mimic many biological and physical properties of the native extracellular matrix (ECM) [1,2], have revealed fundamental insights into how cells interact with their 3D surroundings to direct biological processes [3,4]. Hydrogels amenable to encapsulation of stem cells (SC) have also proven to be important tools in tissue engineering (TE), particularly for the regeneration of cartilage and other soft tissues [[5], [6], [7]], as they can be tuned to direct differentiation as well as entrap secreted proteins. Hydrogels' mechanical and biological characteristics including their stiffness [8] and degradability [9], as well as their viscoelastic properties [10,11], are key drivers of human marrow stromal/mesenchymal stem cell (hMSC) fate. hMSC utilize these cues in concert with those mediated by their own secreted pericellular matrix (PCM) [12] to regulate lineage specification.

Many adult SC populations reside in tissue-specific niches that integrate soluble and non-soluble signals to maintain a delicate balance between self-renewal, quiescence and differentiation [13]. When stimulated by tissue damage, for example, SC often respond by differentiating and secreting appropriate ECM proteins. Hydrogels can be designed to mimic the ECM of native SC niches to allow their residents to either maintain stemness [14] or respond to stimuli [5,9]. However, SC do not exist in isolation in their native niches where they solely respond to cues from their immediate physical surroundings. They also receive instruction from neighboring stem and tissue-specific cells [15]. Indeed, signals generated by neighboring cells govern whether many SC remain quiescent reservoir cells or respond to tissue damage by transforming into transit amplifying cells that proliferate and differentiate to mediate repair [16]. However, the role these non-matrix-mediated stimuli play in *in vitro* systems such as 3D hydrogels is often overlooked. This is particularly important in TE where scaffolds designed to direct SC differentiation often contain high cell densities, which are necessary to produce sufficient ECM. In these contexts, both cell-matrix interactions and contributions from neighboring cells may direct SC response.

To study this, we encapsulated hMSC in hydrogels through a Michael addition between thiol-modified hyaluronic acid (S-HA) and poly(ethylene glycol) diacrylate (PEGDA) [17] (Fig. S1). Cells encapsulated within HA-based hydrogels rely on interactions via surface receptors such as CD44 and CD168 [18] to prevent anoikis, as HA provides no sites for integrin-mediated interactions unless modified chemically with adhesive motifs (Fig. S2). S-HA-PEGDA hydrogels are particularly valuable in examining how the 3D environment regulates SC response, because not only can their physical properties be tuned to mimic those of native SC niches [19], but they also allow for the pericellular retention of ECM proteins secreted by encapsulated cells [12], which is important to understand how SC self-regulate the composition of their own local environment. Here, we held the concentration of S-HA constant and cross-linked hydrogels with either 0.375 or 0.75 relative PEGDA weight. We then used a combination of molecular, imaging and proteomic analyses to examine hMSC response. Our observations demonstrate that high-density (HD) 3D culture in S-HA-PEGDA hydrogels prompts hMSC to take on characteristics of quiescent cells and promotes the formation of a glycoprotein-rich PCM, while low-density (LD) culture favors differentiation. These observations suggest that TE strategies should consider both matrix cues and signaling from neighboring cells in directing hMSC differentiation.

## 2 Materials and methods

### 2.1 Human bone marrow stromal/mesenchymal stem cell (hMSC) isolation, culture and characterization

Human samples were provided by the Imperial College Healthcare Tissue Bank (ICHTB, HTA license 12275) supported by the National Institute for Health Research Biomedical Research Centre at Imperial College Healthcare NHS Trust and Imperial College London. ICHTB is approved by the UK National Research Ethics Service to release human material for research (12/WA/0196). hMSC were generated from bone marrow aspirates (issued from sub-collection R16052) collected from the iliac crest of healthy pediatric donors with informed consent. The total number of nucleated cells was established with a Sysmex SE full blood count analyzer and then 10-25 × 10^6^ cells/636 cm^2^ were plated in CellSTACK^®^ culture chambers (Corning). Cells were cultured in alpha modified Eagle's medium, no nucleosides (αMEM, Gibco) supplemented with 5% human platelet lysate (Stemulate, Cook Medical) under standard culture conditions (37 °C in a humidified atmosphere of 5% CO_2_/95% air). After reaching 90–100% confluency (10–14 days), cells were detached with recombinant trypsin (Roche, DE) and re-seeded at 5000 cells/cm^2^. hMSC were expanded in basal culture medium consisting of αMEM with 10% fetal bovine serum (FBS, Gibco) until passage 7 and regularly checked by flow cytometry to confirm that they expressed CD90, CD105, and CD73 and were negative for CD34 and CD45 [20].

### 2.2 Preparation of hMSC-laden hydrogels

Sodium hyaluronate (Lifecore Biomedical, mean molecular weight 111 kDa) was thiolated as previously described [21]. Thiolated hyaluronic acid (S-HA, with a polymer degree of substitution of 30–40% as determined by Ellman's assay) was sterilized with 25 kGy gamma irradiation using a Gammacell 1000 (Best Theratronics Ltd.). Hydrogels (100 μL) were formed with either 5 × 10^5^ cells/mL (low-density, LD) or 5 × 10^6^ cells/mL (high-density, HD). A single cell suspension in αMEM (8 μL) was mixed with a S-HA solution (8 mg/mL) and a poly(ethylene glycol) diacrylate (PEGDA, ESI-BIO, 3400 Da, 3 or 6 mg/mL, 20 μL) solution in phosphate buffered saline (PBS, without calcium and magnesium, GIBCO) to obtain 1:0.375 or 1:0.75 wt ratios (1:relative weight PEGDA). Cylindrical hMSC-laden hydrogels were formed in polytetrafluoroethylene molds (6 mm diameter) within suspension plates and allowed to cross-link for 2 h under standard culture conditions. After removing the molds, samples were cultured for up to 28 days with basal culture medium supplemented with 1% (v/v) antibiotic-antimycotic solution (Sigma) (1mL/well). Medium was exchanged every 3–4 days. In some experiments, hydrogels were prepared by additionally adding 1% thiol-modified gelatin (Gelin-S, ESI-BIO) or 100 μg/mL fibronectin from bovine plasma (Sigma).

Theoretical calculations of the distance of any hMSC to its ‘nearest neighbor’ if perfectly distributed throughout the hydrogel were carried out according to the approximation by Chandrasekhar [22] whereby hMSC were assumed to be points in 3D space and ‘nearest neighbor’ distance, D, is given as: D = 0.554 P_v_^−1/3^, where *P*_*v*_ is the number of points per unit volume. D approximates the distance between the centers of any 2 cells.

### 2.3 Determination of hMSC viability in S-HA-PEGDA hydrogels

Viability of encapsulated hMSC was quantified using a Real Time-Glo™ MT cell viability assay (Promega) following the manufacturer's instructions. Luminescence was determined on a CLARIOstar^®^ from BMG LABTECH.

### 2.4 Perturbation experiments on encapsulated hMSC

To block hMSC interactions mediated by CD44, integrins that recognize the RGD sequence, and cell-cell interactions mediated by N-cadherins, hMSC were pre-incubated for 45 min at 4 °C with appropriate blocking agents (or controls) in αMEM prior to encapsulation (*n* ≥ 3). CD44-mediated interactions were blocked with 10 μg/mL anti-CD44/H-CAM monoclonal antibody [5F12] (#MA5-12394, Invitrogen) or mouse IgG1, κ isotype control (#MA5-14453, Invitrogen). Both antibodies were concentrated after 3 washes with PBS to remove sodium azide using Amicon 30K MWCO units (Millipore). Integrin-mediated interactions that utilize recognition of the RGD peptide sequence were blocked with 300 μg/mL of RGD sequence-containing peptides (Gly-Arg-Gly-Asp-Ser-Pro, GRGDSP, Bachem, CH). 300 μg/mL of peptide lacking the RGD sequence (Gly-Arg-Ala-Asp-Ser-Pro, GRADSP, Bachem) was used as a control. N-cadherin-mediated interactions were neutralized with anti-N-cadherin monoclonal antibody produced in mouse (clone GC-4) that had been purified from hybridoma cell culture (#C3865, Sigma). A mouse IgG1, κ isotype control (#ab18447, abcam) was used as a control. After encapsulation, basal culture media containing blockers (or controls) were added to appropriate wells.

In some experiments, HD cultures (*n* = 5) were treated with 50 nM paclitaxel (PTX, #ab120143, Abcam). Paclitaxel binds to the N-terminus of β-tubulin inhibiting microtubule disassembly and has been used experimentally to control cell cycle phase [23]. Vehicle controls were cultured in basal culture medium.

### 2.5 RNA isolation and gene expression analyses

hMSC-laden hydrogels were snap frozen in liquid nitrogen and homogenized in Lysing Matrix H tubes (MP Biomedicals) on a FastPrep^®^-24 (MP Biomedicals). RNA was isolated using the RNeasy Mini Kit following the manufacturer's instructions and reverse transcribed into cDNA in two steps. First, 10 μL of RNA, 2 μL of random primers (500 μg/mL), and 2 μL of RNase-free water were incubated at 70 °C for 5 min. Then the reaction mixture was mixed with 1.25 μL of 1 mM PCR Nucleotide Mix, 5 μL M-MLV reverse transcriptase 5× Buffer, 1 μL M-MLV 200 U/μL reverse transcriptase, and 3.75 μL RNase-free water and incubated for 1 h at 42 °C (all from Promega).

qPCR was performed on a Bio-Rad CFX384 Touch™ real-time PCR detection system using 384-Well PCR plates (all Bio-Rad Laboratories Ltd.). Reaction mixtures were prepared with 3 μL of primer mix (forward and reverse; Table S1), 5 μL 2× Brilliant III SYBR Green qPCR Master Mix (Agilent), 0.5 μL of cDNA, and 1.5 μL RNase-free water. A three-step cycle was performed: (1) denaturation at 95 °C for 3 min; (2) annealing/extension at 58 °C for 10 s; and (3) melting from 65 °C to 95 °C at 0.5 °C/step for 5 s. Samples (*n* ≥ 6) and controls (water in place of cDNA) were run in triplicate. The ΔΔCT method was used to quantify fold change in expression (2^−ΔΔCq^) of each gene of interest (GOI) and normalized to the expression of undifferentiated hMSC (day 0, before encapsulation), using stable reference genes (RG) *RPL13a* and *EEF1A1* using the equation: ΔΔCq = [(Cq_GOI,t(x)_ – Cq_RG,t(x)_) – (Cq_GOI,t(0)_ – Cq_RG,t(0)_). Expression values are specific to hydrogel composition, except for chondrogenic genes, where because hydrogel composition did not differentially regulate chondrogenesis in either HD or LD cultures, 1:0.375 and 1:0.75 data are pooled.

### 2.6 Immunofluorescence staining and quantification

After fixing in 4% (w/v) paraformaldehyde in PBS for 20 min, hMSC-laden hydrogels were embedded in 7.5% (w/v) gelatin, 15% (w/v) sucrose and 0.05% (v/v) sodium azide in PBS (all from Sigma). Samples were cryosectioned (10 μm) at −25 °C using a Bright Clinicut 60 cryostat (Bright Instrument Co Ltd.). Before staining, the embedding solution was melted away in PBS at 37 °C. Samples were permeabilized with 0.1% (v/v) Triton-X 100 in PBS for 10 min and blocked for 1 h with 10% (v/v) horse serum in 0.15% (w/v) glycine and 0.2% (w/v) bovine serum albumin (BSA, Sigma) in PBS. Samples stained for versican and lumican were not permeabilized. Primary antibodies were incubated overnight at 4 °C and stained with secondary antibodies for 1 h at room temperature (RT) in the dark. Antibodies were diluted in 1% (v/v) horse serum, 0.15% (w/v) glycine and 0.2% (w/v) BSA in PBS (Table S2). Some samples were stained with 1:100 Phalloidin-Tetramethylrhodamine B isothiocyanate (TRITC) from *Amanita phalloides* (#P1951, Sigma). Samples were washed with PBS between each step. Sections were coverslipped using Fluoroshield™ mounting medium with 4′,6-diamidino-2-phenylindole (DAPI, Sigma). Samples stained for versican, lumican and Ki67 were counterstained for 10 min with 1 μg/mL of DAPI (#D9542, Sigma), coverslipped using PBS, and imaged as intact hydrogels. Controls in the absence of primary antibody, but with appropriate isotype controls were carried out to confirm antibody specificity.

Imaging of collagen type II was carried out on a Nikon A1 confocal laser scanning microscope (Nikon). All other fluorescence imaging was carried out on a Leica DM16000 confocal laser scanning microscope (Leica Microsystems). Z-series with 0.5–2 μm Z-spacing were obtained using sequential acquisition and the Kalman filter mode. A 63× glycerin objective with a numerical aperture of 1.3, or a 40× oil objective with a numerical aperture of 1.25, and a 2048 × 2048 pixel size were used. Detector gains were set to be constant between samples to allow comparison. Staining for collagen type II was analyzed using a pixel intensity-based semi-quantification method with NIS-Elements Advanced Research Analysis 4.30.01 64-bit software after removing non-specific events generated by noise and background. The sum signal intensity was measured using regions of interest on the 488 channel in 3D images of at least 20 cells per experimental condition. The percentage of hMSC stained with Ki67 was quantified using 250 cells in LD cultures and 500–600 cells in HD cultures. Images show 3D projections generated with the Image J plugin 3D viewer (National Institutes of Health).

### 2.7 Imaging and quantification of protein synthesis and cell volume

hMSC were prepared in high glucose DMEM without glutamine, methionine, or cystine (#21013024, Life Technologies) supplemented with 4 mM l-glutamine, 0.201 mM l-cystine (#C7602, Sigma), 1 mM sodium pyruvate (Fisher Scientific), 50 μg/mL ascorbate 2-phosphate (Sigma), and 40 μg/mL proline (Sigma). Cell-laden hydrogels were cultured for 72 h in medium supplemented with 1.25 mg/mL BSA, 0.1% insulin-transferrin-selenium (Thermo Fisher Scientific), 1% (v/v) antibiotic-antimycotic solution and with either 0.1 mM l-methionine (#M5308, Sigma; control media) or 0.1 mM l-homopropargylglycine (HPG, #C10186, Molecular Probes; labeling media). This approach allows the methionine analog HPG to be incorporated into proteins as they are synthesized [24]. HPG was tagged using a click-IT HPG Alexa Fluor 488 protein synthesis assay kit (#C10428, Molecular Probes) following the manufacturer's instructions. Samples were fixed in 4% (w/v) paraformaldehyde in PBS for 20 min, rinsed twice with PBS, and incubated for 1 h with the labeling solution, which included an Alexa Fluor 488 azide. Nuclei were labeled with NuclearMask™ in PBS for 15 min, and samples were washed thrice with PBS before imaging. Controls were treated identically to labeled samples.

Samples were coverslipped using PBS and imaged as intact gels on a Leica DM16000 confocal laser scanning microscope. Differential interference contrast (DIC) imaging was used to delineate the cell outline. Detector gains were set to be constant between samples to allow for comparison. Z-series with 2 μm Z-spacing were obtained using sequential acquisition and the Kalman filter mode. A 40× oil objective with a numerical aperture of 1.25 and a 2048 × 2048 pixel size was used to capture images. A pixel intensity-based semi-quantification of labeled proteins was performed with Image J after removing non-specific events generated by noise and background and using a constant threshold defined according to control images. The sum integrated density of total produced protein per cell was measured using regions of interest in the 488 channel in 3D images (only when the entire cell and its respective PCM were captured). DIC images of the outline of the cell containing contiguous saturated pixels in the 488 channel allowed for measurement of cell volume in Image J. Values are reported for at least 20 cells per condition. Images show 3D projections obtained using the Image J plugin 3D viewer.

### 2.8 Evaluation of paracrine effects on hMSC differentiation

Two sets of hMSC-laden 1:0.75 hydrogels, at low density (5 hydrogels of 100 μL at 5 × 10^5^ cells/mL) and at high density (1 hydrogel of 50 μL at 5 × 10^6^ cells/mL), were prepared over two consecutive days (*n* = 5). This experimental design ensured the same number of cells and cell culture medium volume in both LD and HD conditions. The first set of hydrogels (created on the first day) was used to obtain conditioned media (2 mL) to treat the second set of hydrogels (created on the second day). The donor groups were given fresh basal culture media daily and their conditioned media was transferred to recipient hydrogels. Media were transferred such that LD cultures received media that had been conditioned on HD cultures and *vice versa*. After 72 h, recipient hydrogels were collected for gene expression analyses and the donors were used as controls.

### 2.9 Proteomic analysis of hMSC pericellular matrix formation

hMSC-laden hydrogels (*n* = 3) were prepared as described above, except the cell suspension was washed extensively with “heavy” SILAC medium (SILAC αMEM, no nucleosides, no lipoic acid prepared in-house and supplemented with 0.1 mg/mL heavy isotopically-labeled lysine and arginine, #CNLM-291-H, #CNLM-539-H, Cambridge Isotope Laboratories, Inc.) prior to encapsulation. LD and HD hydrogel cultures were cultured for 72 h in “heavy” SILAC medium with 10% dialyzed FBS (against PBS using Amicon 3K MWCO units, Millipore) and 1% antibiotic-antimycotic solution. Samples were decellularized with 0.5% Triton X-100 and 20 mM ammonium hydroxide in PBS for 90 min at RT followed by 3 washes with PBS. The efficiency of hydrogel decellularization was confirmed by staining nuclei with DAPI and actin with Phalloidin-TRITC (data not shown).

#### 2.9.1 In-hydrogel digestion

Hydrogels were incubated for 10 min at RT with 100 μL acetonitrile (Optima, LC/MS Grade, Fisher Chemical) and then transferred to 2 mL tubes and the acetonitrile removed. 100 ng/μL lyophilized trypsin (Sequencing Grade Modified Trypsin, Promega) in 50 mM acetic acid was diluted ten-fold in 100 mM ammonium bicarbonate to give a 10 ng/μL working solution. 50 ng trypsin was added to each hydrogel and allowed to absorb for 30 min at RT. 100 μL 100 mM ammonium bicarbonate was added, and the hydrogel was incubated overnight at 37 °C on a bench top mixer. Tubes were centrifuged (2000 rpm, 3 min, 25 °C) and the supernatant was transferred to new 2 mL tubes. 30 μL acetonitrile was added to the hydrogel and incubated for an additional 5 min at RT and then centrifuged (2000 rpm, 3 min, 25 °C) again, and the supernatant combined with the previous supernatant creating the final sample. Samples were vacuum centrifuged for 30 min, and a C18 reversed phase clean-up was performed using in-house prepared StageTips [25]. Peptides were eluted from the StageTip with 80% acetonitrile/5% trifluoroacetic acid (TFA) and vacuum centrifuged to dryness.

#### 2.9.2 Mass spectrometry (MS) analysis

Peptides were re-suspended in 15 μL 0.1% TFA, sonicated for 10 min and centrifuged (14,000 rpm, 3 min, 25 °C). 5 μL of supernatant was transferred to a glass autosampler vial containing 45 μL of 0.1% TFA to give a 1:10 dilution (dilution determined based on preliminary experiments). 10 μL sample was then analyzed in technical duplicate using a ThermoFisher Scientific Fusion Lumos mass spectrometer coupled to an UltiMate 3000 HPLC system for on-line liquid chromatographic separation. The sample was loaded onto a C18 trap column (ThermoFisher Scientific Acclaim PepMap 100; 75 μM × 2 cm) then transferred onto a C18 reversed-phase column (ThermoFisher Scientific PepMap RSLC; 50 cm length, 75 μm inner diameter). Peptides were eluted with a linear gradient of 2–27.5% buffer B (75% acetonitrile, 20% water, 0.1% formic acid, 5% DMSO) at a flow rate of 275 nL/min over 107 min, followed by a second linear gradient of 27.5–40% buffer B over 10 min. MS spectra were acquired in the orbitrap at 120,000 resolution, with scan range of 350–1500 *m*/*z*. A maximum injection time of 50 ms, an AGC target value of 4E5, a 40% RF lens setting and a 60 s dynamic exclusion were included. Precursor ions with charge states z = 2–8^+^ were selected for MS/MS collision-induced dissociation fragmentation (35% energy) and data acquired in the ion trap. A maximum injection time of 300 ms and an AGC target value of 2E3 were included. A “Universal Method” [26] was adopted which aims to maximize detection of peptides irrespective of complexity and sample abundance.

#### 2.9.3 Proteomic data analysis

Data were analyzed with MaxQuant software (version 1.3.0.5). MaxQuant analyzed samples by selecting a multiplicity of 2 and heavy labels Arg10 and Lys8. MS/MS spectra were searched against an UniProtKB human protein database (downloaded May 2017; 159,743 entries) using the Andromeda search engine, including common protein contaminants in the search. Trypsin enzyme was selected (C-terminal cleavage of arginine and lysine residues) and a maximum of 2 missed cleavages was permitted. Carbamidomethylation of cysteine was set as a fixed modification; N-terminal protein acetylation and methionine oxidation were set as variable modifications. For all other MaxQuant parameters, we maintained default settings, including a peptide false discovery rate of 0.01. The resulting proteinGroups.txt file was opened in Microsoft Office Excel 2016. The percentage heavy label incorporation was calculated for each protein using the formula (Ratio H/L)/(1 + Ratio H/L)*100. The file was saved as a .txt file and opened in Perseus (version 1.4.0.2). For each sample, we set a minimum “Ratio H/L count” threshold of 3 for both technical duplicates. The “Ratio H/L count” refers to the number of peptide quantification events used for protein quantification. Gene Ontology annotation was performed to identify ECM proteins.

### 2.10 Statistical analyses

All experiments and analysis were performed in a blinded manner. All results are presented as means + SD of the biological replicates except for fluorescence intensity quantification measurements, which are shown as medians + SEM. Statistical analyses to compare measurements on LD and HD cultures or between treated and control groups were performed using a non-parametric two-tailed Mann-Whitney test. Statistical analyses of hMSC viability in HD cultures over time was carried out using a non-parametric Kruskal-Wallis test followed by Dunn's multiple comparison test. Comparisons of Ki67 staining of hMSC were performed using a Fisher's exact test. All statistical analyses were carried out using GraphPad Prism version 7 for Windows (GraphPad Software). *P* values are indicated in figure captions.

## 3 Results

### 3.1 HD hMSC cultures do not rely extensively on HA, many integrin- or N-cadherin-mediated interactions for viability

**Fig. 1:**
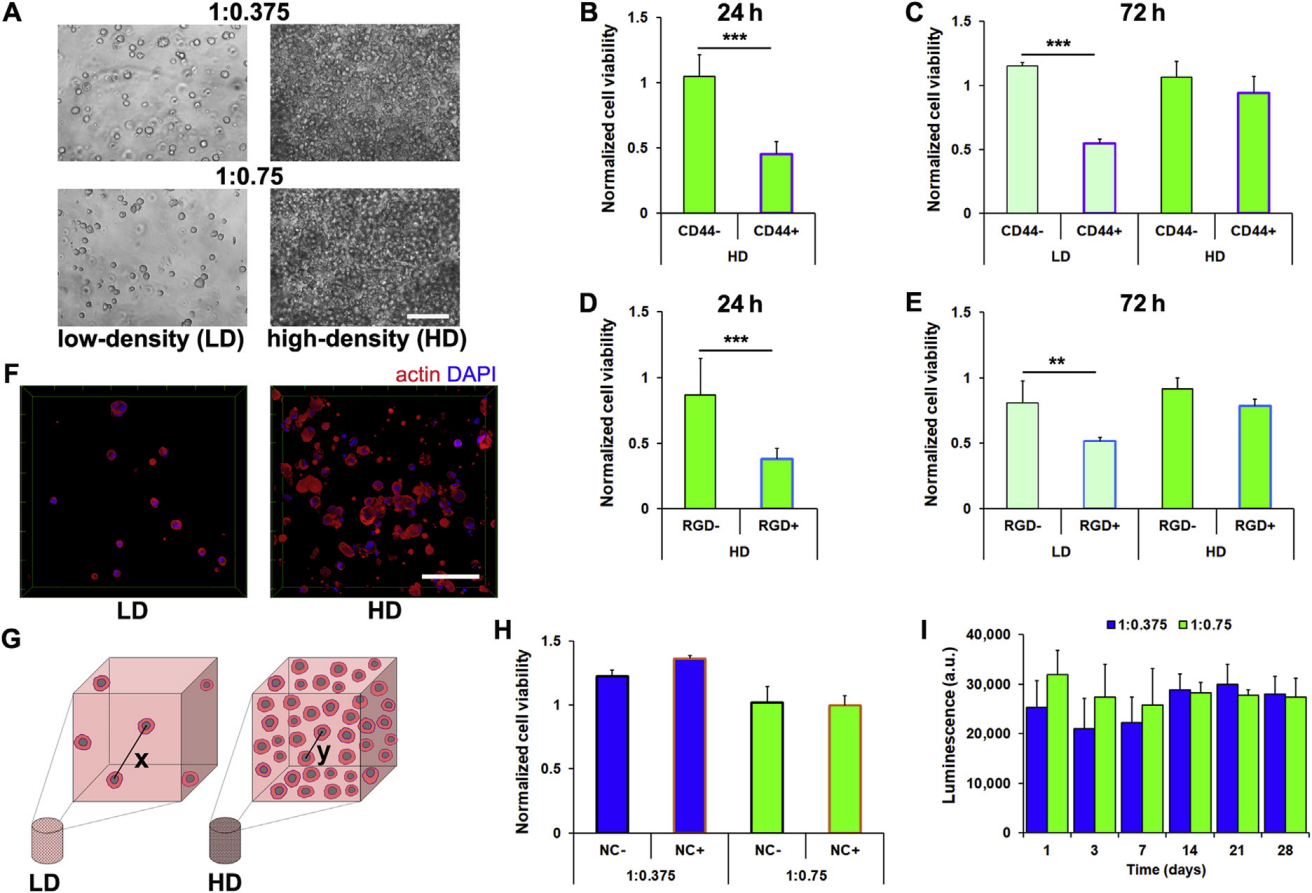
**HD cultures do not rely on matrix cues or N-cadherin interactions to prevent anoikis.** (**A**) Representative micrographs of hMSC (5 × 10^5^ cells/mL (LD), 5 × 10^6^ cells/mL (HD)) in 1:0.375 and 1:0.75 S-HA-PEGDA hydrogels after 24 h. (**B**) Viability of HD cultures of hMSC encapsulated within 1:0.75 hydrogels for 24 h and treated with anti-CD44 (CD44^+^) antibodies or isotype controls (CD44^−^) and normalized to vehicle controls, and (**C**) in both HD and LD cultures for 72 h (*n* = 3, ***P < 0.001). (**D**) Viability of HD cultures of hMSC in 1:0.75 hydrogels for 24 h and treated with RGD sequence-containing peptides (RGD+) or scrambled peptides (RGD-) and normalized to vehicle controls, and (**E**) in both HD and LD for 72 h (*n* = 3, **P < 0.01, ***P < 0.001). (**F**) Representative micrographs of hMSC in LD and HD cultures for 72 h in 1:0.75 hydrogels stained with Phalloidin-TRITC. (**G**) Schematic highlighting theoretical distances between any two ‘nearest neighbor’ cells, where x = 69.8 μm and y = 32.4 μm. (**H**) Viability of LD and HD cultures of hMSC encapsulated in 1:0.75 hydrogels for 72 h and treated with anti-N-cadherin antibody (NC+) or isotype controls (NC-) and normalized to vehicle controls (*n* = 3). (**I**) Viability of HD cultures of hMSC in 1:0.375 and 1:0.75 S-HA-PEGDA hydrogels after up to 28 days (*n* = 3). In (A) and (F) scale bar = 100 μm. Plots show mean + SD. In (B)–(E) and (H) a two-tailed Mann-Whitney test, and in (I) a Kruskal-Wallis test followed by Dunn's Multiple Comparison were used to detect statistical significance.

To begin to explore the relative contributions provided by 3D culture conditions in which matrix cues versus signaling between neighboring cells played dominant roles in directing response, we encapsulated single-cell suspensions of hMSC in S-HA-PEGDA hydrogels at densities of either 5 × 10^5^ (low-density, LD) or 5 × 10^6^ (high-density, HD) cells/mL (Fig. 1A). In line with the expected role of HA in mediating cell viability in hydrogels that have not been modified with adhesive motifs, blocking hMSC-HA interactions in HD cultures with anti-CD44 antibodies resulted in a significant decrease in cell viability after 24 h (Fig. 1B). However, unlike in LD cultures where this effect persisted, blocking with anti-CD44 antibodies for 72 h in HD cultures had no effect on viability compared to isotype antibody-treated controls (Fig. 1C). LD hMSC cultures within S-HA-PEGDA hydrogels secrete proteins pericellularly, which they rely on for viability [12]. In HD cultures, the addition of RGD-sequence containing peptides, which block many integrin-mediated interactions, similarly significantly decreased hMSC viability over the first 24 h after encapsulation (Fig. 1D). However, they had no effect on the viability of HD cultures after 72 h when compared to scrambled peptide-treated controls. This contrasted with observations in LD cultures in which RGD-sequence containing peptides continued to negatively affect hMSC viability (Fig. 1E).

As HD hMSC cultures appeared not to rely on cell-matrix interactions for survival to the same extent as LD cultures, we next asked if direct cell-cell interactions could have been playing a role, as MSC are known to remain viable as spheroids [27,28]. 4 h after encapsulation, cultures contained single cells with round morphologies (Fig. S3). After 72 h, hMSC in HD cultures were observed in closer proximity to one another compared to in LD cultures, in which they were almost always far from their closest neighbors; however, hMSC in HD cultures did not aggregate or form spheroids or pellets (Fig. 1F), as has been observed in suspension culture [27,28]. Theoretical calculations [22] showed that if perfectly distributed, centers of hMSC in LD cultures would be 69.8 μm from their ‘nearest neighbor’, while in HD cultures they would only be separated by 32.4 μm (Fig. 1G). hMSC aggregation is highly dependent on cadherins, transmembrane proteins that mediate many cell-cell interactions, and particularly on N-cadherin, which plays important roles in MSC differentiation [29]. However, treating HD cultures with anti-N-cadherin antibody had no effect on viability (Fig. 1H), nor did HD culture stimulate proliferation, as we did not detect differences in the total number of viable cells after up to 28 days (Fig. 1I). Instead, these observations suggest that although hMSC in HD cultures did not rely on N-cadherin-mediated interactions for viability; and moreover, as viability did not depend strongly on interactions with HA (mediated through CD44) nor many integrin-mediated interactions with a secreted PCM, they suggest that hMSC in HD cultures were either not as susceptible to anoikis as LD cultures or that they relied on alternative interactions to prevent it.

### 3.2 Neither matrix cues nor N-cadherin-mediated interactions direct hMSC differentiation in HD cultures

**Fig. 2:**
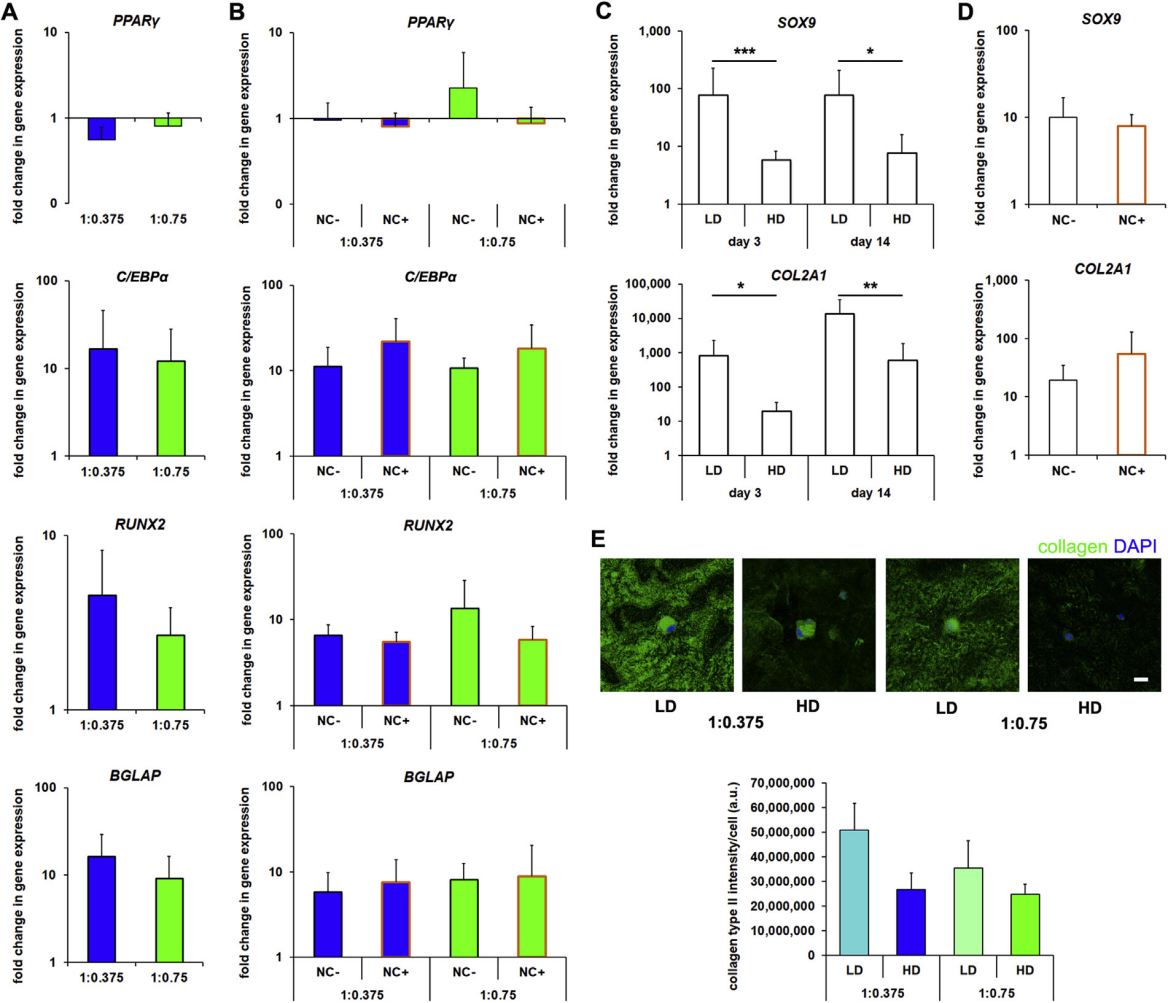
**HD cultures do not differentiate in response to hydrogel cues**. (**A**) Gene expression analyses (normalized to undifferentiated controls) for markers of adipogenesis (*PPARγ*, *C/EPBα*) and osteogenesis (*RUNX2*, *BGLAP*) in HD cultures in 1:0.375 and 1:0.75 S-HA-PEGDA hydrogels after 72 h in culture (*n* ≥ 6), or (**B**) treated with anti-N-cadherin antibody (NC+, outlined orange) or isotype controls (NC-) (*n* ≥ 6). (**C**) Gene expression analyses for markers of chondrogenesis (*SOX9*, *COL2A1*) in LD and HD cultures after 3 or 14 days (*n* ≥ 12, *P < 0.05, **P < 0.01, ***P < 0.001), or (**D**) in HD cultures treated for 72 h with anti-N-cadherin antibody (NC+, outlined orange) or isotype controls (NC-) (*n* ≥ 16). (**E**) Representative micrographs of LD and HD cultures after 28 days and stained for collagen type II. Plot shows a pixel intensity-based semi-quantification of collagen type II produced per cell (*n* ≥ 20). Scale bar = 20 μm. In (A)–(D) bars show mean + SD, and in (E) bars show median + SEM. In (A)–(D) a two-tailed Mann-Whitney test was used to detect statistical significance.

In LD cultures, 1:0.375 S-HA:PEGDA hydrogels promote a bias towards adipogenesis and 1:0.75 towards osteogenesis [12]. Therefore, we hypothesized that hydrogel composition would similarly drive hMSC lineage specification in HD cultures. However, when we examined expression of markers for adipogenic and osteogenic fates by qPCR, we found no significant effect of hydrogel composition (Fig. 2A). Therefore, we next hypothesized that direct cell-cell interactions may have instructed hMSC response, and that by blocking them we could prompt hMSC to adopt hydrogel composition-dependent fates. However, blocking cell-cell interactions in HD cultures with antibody against N-cadherin had no effect on expression of a panel of osteogenic and adipogenic genes (Fig. 2B).

As hMSC in HD cultures did not upregulate adipogenic/osteogenic genes in response to hydrogel composition, we next speculated that hMSC might be undergoing chondrogenic differentiation as both HA-based hydrogels and cell-cell interactions (driven by N-cadherin-mediated interactions) are known drivers of chondrogenesis [5,6,30]. However, when we examined chondrogenic genes, we found that *COL2A1* and *SOX9* expression were lower in HD compared to LD cultures (Fig. 2C). Treating hMSC with anti-N-cadherin antibody had no effect on chondrogenic gene expression under these conditions (Fig. 2D). Moreover, LD cultures showed more extensive positive immunostaining for collagen type II than HD cultures (Fig. 2E). Taken together, these data suggest that HD cultures were not driving chondrogenesis; and more globally, that while in LD cultures, hydrogel composition strongly influenced hMSC fate, in HD cultures, it had little to no effect. Indeed, it seemed that hMSC response in HD cultures was guided not by hydrogel composition, but rather by the presence of neighboring cells, although this was not via direct N-cadherin-mediated interactions.

### 3.3 HD cultures produce less protein and release factors that diminish matrix-driven differentiation

**Fig. 3:**
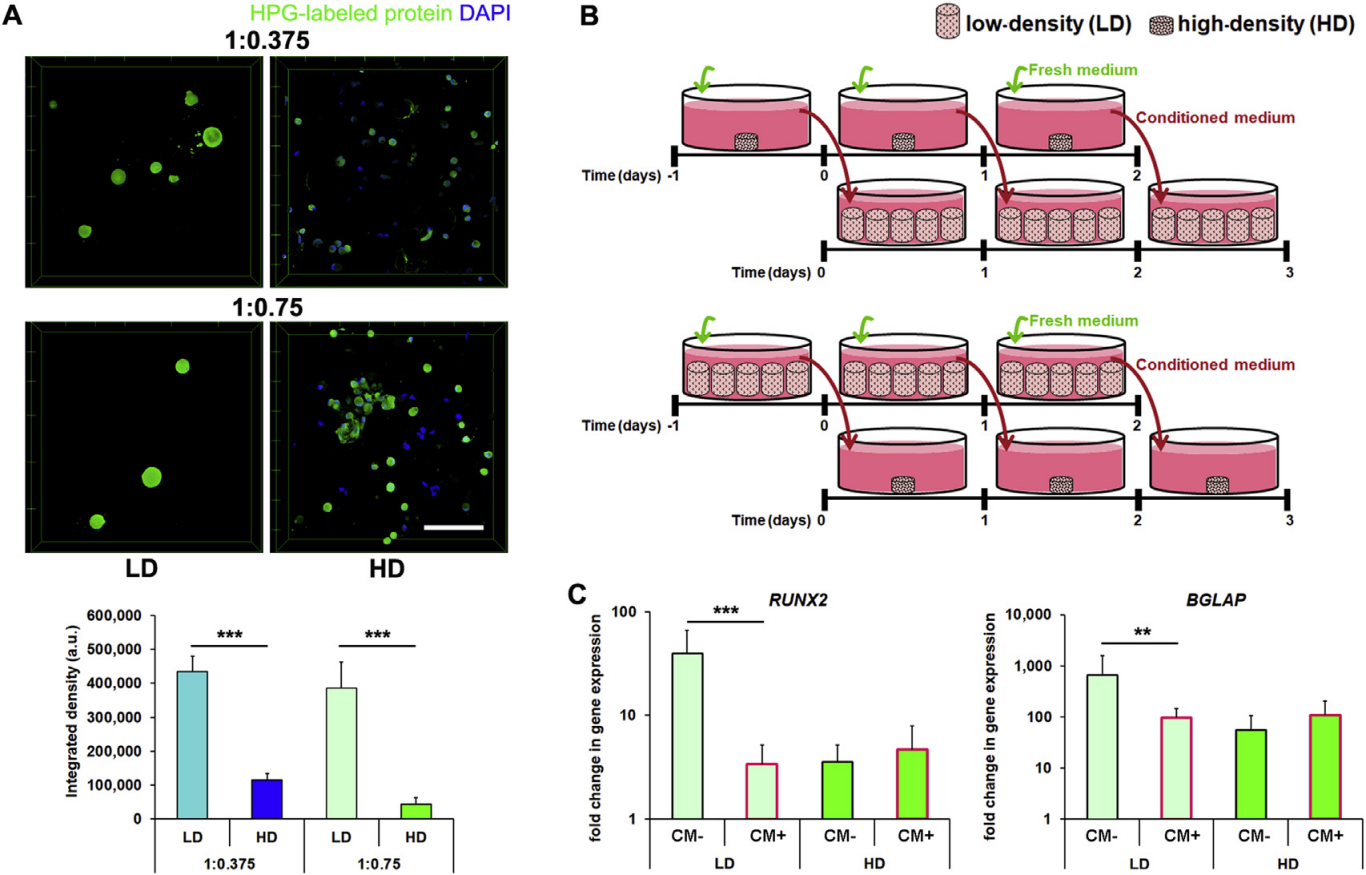
**HD cultures produce less protein and release factors that downregulate hMSC differentiation**. (**A**) Representative micrographs of HPG-labeled proteins produced by LD and HD cultures of hMSC in 1:0.375 and 1:0.75 hydrogels after 72 h. Scale bar = 100 μm. Total protein produced by hMSC was determined by quantifying the integrated density of fluorescence labeling on a per cell basis (*n* ≥ 20, ***P < 0.001). (**B**) Schematic of experimental design to examine the role of paracrine signaling. Either conditioned medium (CM) from HD cultures was placed on LD cultures, or CM from LD cultures was placed on HD cultures such that the total number of cells (2.5 × 10^6^ cells/well) and volume of culture media was kept constant. CM was transferred every 24 h for a total of 72 h. (**C**) Gene expression analyses (normalized to undifferentiated controls) for markers of osteogenesis (*RUNX2*, *BGLAP*) in LD and HD cultures 72 h after encapsulation in 1:0.75 hydrogels. hMSC-laden hydrogels were cultured with basal medium (CM-, control) or treated with CM (CM+, outlined pink) (*n* ≥ 6, **P < 0.01, ***P < 0.001). In (A) bars show median + SEM and in (C) bars show mean + SD. In (A) and (C) a two-tailed Mann-Whitney test was used to detect statistical significance.

As we have previously shown that at LD, hMSC can secrete a proteinaceous PCM that drives their differentiation in S-HA-PEGDA hydrogels [12], we next hypothesized that differential observations in HD and LD cultures could similarly be attributed to PCM formation. Therefore, we employed fluorescent non-canonical amino acid tagging (FUNCAT) to label intracellular and secreted PCM proteins produced by hMSC while encapsulated in hydrogels. This technique replaces a canonical amino acid (methionine) with the non-canonical analog l-homopropargylglycine (HPG), which can then be identified with a fluorescent tag using a simple click chemistry [24]. LD hMSC cultures assembled an extensive PCM; however, HD cultures formed very little (Fig. 3A and Fig. S4). Quantification of the total amount of protein produced confirmed that HD cultures produced significantly less protein per cell than LD cultures.

As density itself seemed to guide hMSC response in HD cultures, we next hypothesized that paracrine effects mediated by soluble factors released from cells could have played a role. Therefore, we designed an indirect co-culture experiment to examine their role on hMSC differentiation, holding the number of hMSC and volume of culture media in all conditions constant (Fig. 3B). LD 1:0.75 hydrogels drive osteogenesis [12], but HD cultures do not. When we placed conditioned medium (CM) from HD 1:0.75 hydrogels on LD 1:0.75 cultures, we observed decreased *RUNX2* and *BGLAP* expression. However, when we placed CM from LD cultures on HD cultures, we observed no effect (Fig. 3C). Media conditioned on HD cultures similarly decreased chondrogenic gene expression in LD cultures, but LD-culture CM had no effect on the chondrogenesis of HD cultures (Fig. S5). These observations confirmed that in contrast to LD cultures, hMSC in HD cultures appeared to produce factors which could override hMSC's hydrogel composition-dependent response and discourage differentiation.

### 3.4 HD cultures drive hMSC quiescence and formation of a glycoprotein-rich PCM

**Fig. 4:**
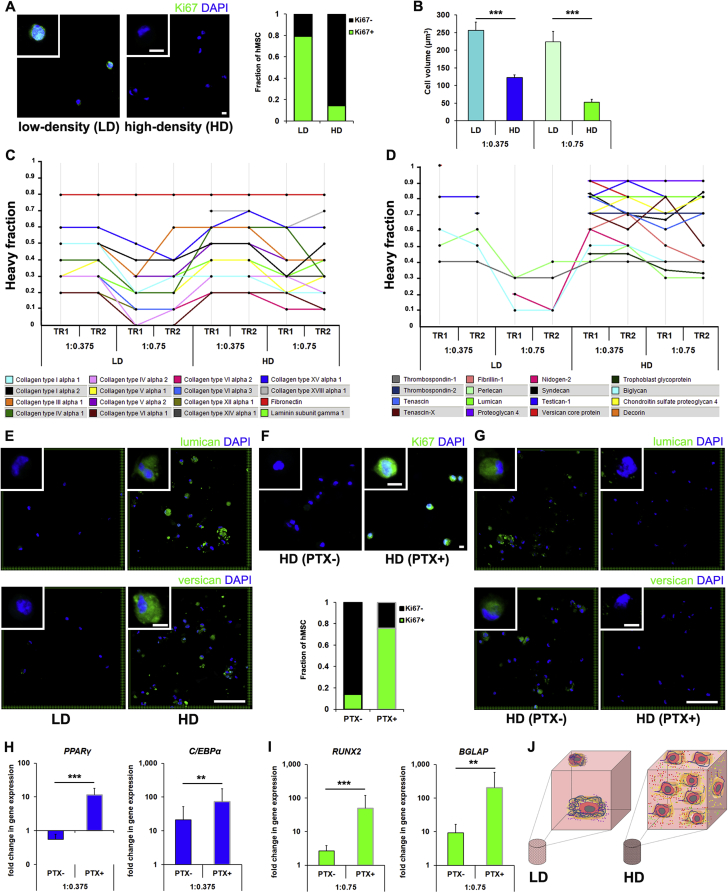
**HD cultures secrete a glycoprotein-rich PCM and take on characteristics of quiescent cells**. (**A**) Representative micrographs of Ki67 staining of hMSC in 1:0.75 hydrogels after 72 h. Significantly more hMSC were positive for Ki67 in LD (*n* = 250) than HD (*n* ≥ 500) cultures (*P* < 0.001). (**B**) Quantification of hMSC volume in LD and HD cultures (*n* ≥ 20, ***P < 0.001). (**C**) Profile plots showing the heavy fraction of a selection of structural proteins, or (**D**) glycoproteins synthesized by LD and HD cultures of hMSC encapsulated in 1:0.375 and 1:0.75 S-HA-PEGDA hydrogels (two technical replicates, TR) after 72 h in culture. (**E**) Representative micrographs showing staining for lumican and versican secreted by hMSC in LD and HD cultures in 1:0.375 hydrogels after 72 h. (**F**) Representative micrographs showing Ki67 staining of hMSC in HD cultures within 1:0.75 hydrogels after treatment with paclitaxel (PTX+) or vehicle control (PTX-) for 72 h. Significantly more hMSC were positive for Ki67 in the presence of paclitaxel than vehicle controls (*n* ≥ 500, *P* < 0.001). (**G**) Representative micrographs showing staining for lumican and versican secreted by hMSC in 1:0.75 hydrogels treated with paclitaxel or the vehicle control after 72 h. (**H**) Gene expression analyses (normalized to undifferentiated controls) in HD cultures of hMSC for markers of adipogenesis (*PPARγ*, *C/EPBα*) in 1:0.375, and (**I**) osteogenesis (*RUNX2*, *BGLAP*) in 1:0.75 hydrogels 72 h after encapsulation. Cultures were treated with paclitaxel (outlined grey) or with the vehicle control (*n* ≥ 8, **P < 0.01, ***P < 0.001). (**J**) Schematic highlighting potential means by which HD cultures could regulate hMSC response compared to LD cultures. In LD cultures, hMSC rely on cues from the hydrogel, secrete PCM and interact with it to differentiate. HD culture may drive quiescence via an indirect effect mediated by the formation of interacting PCM and their sequestration of signaling molecules. In (A) and (F) scale bar = 10 μm. In (E) and (G) scale bar = 100 μm, insets = 10 μm. In (A) and (F) a Fisher's exact test was used to detect statistical significance. In (B), and (H)–(I) plots show mean + SD and a two-tailed Mann-Whitney test was used to detect statistical significance.

Most adult SC exist *in vivo* in state of quiescence marked by low metabolic activity, persistence in the G_0_ phase of the cell cycle, and maintenance of multipotency [13]. SC also often have smaller cell volumes [31] and their stemness has been associated with a reduced susceptibility to anoikis [32]. The identification of definitive MSC *in vivo* is controversial [33,34], but many have speculated that MSC are quiescent in the bone marrow [13]. They also appear to respond like other quiescent adult SC to injury or placement on tissue culture plastic by taking on characteristics of transit amplifying cells [35,36]. Therefore, as HD cultures did not show hydrogel composition-dependent differentiation, had lower overall protein production (suggesting they were less metabolically active), were less susceptible than LD cultures to anoikis, and appeared to secrete factors which suppressed differentiation, we next hypothesized that HD culture stimulated hMSC to become quiescent. As one of the hallmarks of quiescence is temporary exit from the cell cycle, we stained cultures for Ki67 to determine the percentage of encapsulated hMSC in active phases of the cell cycle. We observed that in HD cultures, significantly more hMSC were negative for Ki67 than in LD cultures (Fig. 4A). Moreover, quantification of total cell volume, which in muscle satellite cells is associated with quiescent versus activated phenotypes [31], showed that hMSC in HD cultures were significantly smaller than those in LD cultures (Fig. 4B).

Another characteristic of adult SC niches is the presence of specific pericellular ECM proteins that are thought to help maintain stemness, likely by sequestering signaling molecules [37,38]. Therefore, we next characterized the composition of the ECM secreted by LD and HD cultures and retained in hydrogels by carrying out a stable isotope labeling by amino acids in cell culture (SILAC)-based proteomic analysis. After decellularization, we identified 2046 proteins (with a 1% false discovery rate) by mass spectrometry. Comparing the differential incorporation of the heavy amino acid label in LD and HD cultures, we first observed that many structural proteins known to be secreted by hMSC [39], including collagens and fibronectin, showed relatively similar incorporation of the heavy label in both groups (Fig. 4C + Data file S1). This precluded attributing differences in heavy label incorporation in LD and HD cultures as differentially affecting the entire secreted proteome. However, many glycoproteins, including lumican, versican, tenascin, and proteoglycan 4, among others, showed robust incorporation of the heavy label in HD cultures, but in LD cultures incorporation was minimal or we could not calculate a ratio (Fig. 4D). Similarly, proteins known to play functional or regulatory roles in adult SC niches, including growth factors and their binding proteins, showed high levels of incorporation in HD cultures (Fig. S6). This suggested that although hMSC in both LD and HD cultures synthesized a range of ECM proteins, HD cultures preferentially secreted glycoproteins and proteins supportive of adult SC niches. To confirm our SILAC-based observations, we carried out immunostaining for lumican and versican, members of the small leucine-rich proteoglycan and lectican families of proteoglycans, respectively (Fig. 4E). In HD cultures, we observed positive extracellular staining for both lumican and versican, but in LD cultures, we detected little to none. Absent from the list of glycoproteins that were differentially secreted by HD versus LD cultures was the cartilage-specific glycoprotein aggrecan, supporting our observations that HD cultures did not promote chondrogenesis.

### 3.5 Preventing quiescence precludes the formation of a glycoprotein-rich PCM and prompts hMSC to undergo hydrogel composition-driven differentiation

As hMSC in HD cultures appeared to adopt quiescent phenotypes, we next asked if we could reverse the inhibitory effects of high density on differentiation by preventing hMSC from entering G_0_. Microtubule dynamics play important roles in the cell cycle [40]. Therefore, we hypothesized that the microtubule depolymerizing agent paclitaxel could prompt hMSC to remain in an active phase of the cell cycle. Treatment of encapsulated hMSC with 50 nM paclitaxel did not affect viability (Fig. S7). However, when we stained for Ki67, we observed that HD cultures stained positively for Ki67 (Fig. 4F) at levels no different to those in LD cultures (*P* > 0.05), confirming that paclitaxel prevented hMSC from exiting the cell cycle. Immunostaining for lumican and versican was similarly reduced in HD cultures after treatment (Fig. 4G), suggesting that preventing hMSC from moving into G_0_ precluded the secretion a glycoprotein-rich PCM. Moreover, expression analyses showed that paclitaxel treatment of HD cultures increased expression of adipogenic genes in 1:0.375 hydrogels and osteogenic genes in 1:0.75 hydrogels (Fig. 4H–I), and drove a general increase in chondrogenesis (Fig. S8) similar to that observed in LD cultures [12]. In short, by preventing hMSC from moving out of the cell cycle, we were able to halt the secretion of a glycoprotein-rich PCM and force hMSC to adopt lineages as directed by hydrogel composition.

## 4 Discussion

Unlike tissue cells that are often terminally differentiated and thus cannot re-enter the cell cycle, many adult SC are quiescent in their native niches, but can respond to stimuli by re-entering the cell cycle, differentiating and producing abundant ECM. Here, we show that HD cultures of hMSC encapsulated within S-HA-PEGDA hydrogels take on characteristics of quiescent cells, whereas in LD cultures, they remain in an active phase of the cell cycle, respond to cues provided by their 3D environment and differentiate. S-HA-PEGDA hydrogels are soft immediately upon formation, and continue to stiffen for hours [19]. Culturing hMSC on soft surfaces (250 Pa) has been shown to drive quiescence [41]. These observations suggest that the soft conditions hMSC experience immediately upon encapsulation may have created a bias towards quiescence, which combined with signaling from neighboring cells, HD cultures maintained by secreting a glycoprotein-rich PCM. However, in LD cultures, the initial bias towards quiescence may have been countered by a lack of signaling from neighboring cells, which prompted hMSC to instead re-enter the cell cycle, where they differentiated in response to hydrogel cues.

By preventing HD hMSC cultures from taking on characteristics of quiescent cells (lack of staining for Ki67), we demonstrated that they no longer formed a PCM rich in glycoproteins such as lumican and versican, but instead differentiated as instructed by the hydrogel. It is not possible to speculate whether a lack of signaling from neighboring cells in LD cultures directed hMSC into an active phase of the cell cycle whereby they differentiated, or if LD culture prompted secretion of specific PCM proteins, which subsequently directed cell cycle phase, but both possibilities remain options. Cells are known to adopt quiescent phenotypes in response to stimuli including nutrient deprivation, contact inhibition/growth restriction, and anchorage deprivation [13]. It is unlikely that these factors played a significant role here, however. Paclitaxel prompted hMSC in HD cultures to differentiate, precluding a nutrient deprivation-driven effect. Moreover, hMSC in both HD and LD cultures were equally deprived of adhesive ligands immediately upon encapsulation, ruling out a role for anchorage deprivation. Contact inhibition also appears to be an unlikely explanation because even in HD cultures, we rarely observed hMSC in intimate contact with other cells.

Our SILAC-based proteomic analysis showed that HD culture stimulated the formation of a glycoprotein-rich PCM. Glycoproteins play important roles in regulating multipotency in adult SC niches by sequestering growth factors and regulating their signaling [42,43]. Indeed, decorin is a critical binder of TGFβ1, rendering it inactive [44]. In the absence of glycoproteins, TGFβ1 binds to its receptor on MSC [45] and activates its signaling pathway. MSC are also known to secrete ECM that can regulate their own multipotency. Culturing MSC on a self-secreted matrix composed of structural and glycoproteins, which bind growth factors such as BMP-2, inhibited their spontaneous differentiation [46]. In addition to glycoproteins, our proteomic screen also showed that HD cultures synthesized heavy-labeled proteins known to be functional or regulatory within adult SC niches. For example, HD cultures produced TGFβ1 and latent-TGFβ-binding protein 1 (LTBP1), which binds latent TGFβs to the ECM. Similarly, HD cultures showed high levels of incorporation of the heavy label into matrix metalloproteinases-2 and -14, which play important roles in activating TGFβ signaling [47].

We observed that hMSC in HD cultures released factors that suppressed differentiation in LD cultures. This is in keeping with previous observations that high densities achieved by culturing hMSC in spheroids [27,28], or seeding rat MSC on microporous alginate scaffolds [48], enhanced paracrine signaling. In previous studies, direct cell-cell interactions were essential as anti-N-cadherin antibody precluded the paracrine effect [48]. We were not able to demonstrate a role for direct cell-cell interactions here. Moreover, whereas others observed that MSC paracrine signaling drives differentiation [48], in our S-HA-PEGDA hydrogels, paracrine signaling appeared to inhibit differentiation. Such differences may be explained by a mechano-transduction effect. Both spheroid culture and microporous scaffolds not only allow for cell-cell interactions, but also spread cell morphologies. In S-HA-PEGDA hydrogels, hMSC remain round, even at high densities. Round morphologies may be more akin to those MSC adopt in their *in vivo* niche, as Nestin + MSC are spherical in the bone marrow [33]. Moreover, while the presence of neighboring cells clearly played a role in driving hMSC response, our analyses have not definitively identified how this effect was mediated. CM suppressed expression of genes associated with differentiation; however, in these experiments we held the total number of cells and culture media volume constant, suggesting that secreted factor concentration could not completely explain our results. Soluble factors also diffuse easily through S-HA-PEGDA hydrogels [12], making it unlikely that diffusion-driven secreted factor gradients formed around cells. Instead, our observations point towards an indirect effect mediated by interactions between PCM, signaling molecule sequestration, or other intercellular communication mechanisms (Fig. 4J). Indeed, cellular protrusions, perhaps via tunneling nanotubes and various forms of extracellular vesicles have recently been shown to play profound roles in mediating communication between cells that are not physically in contact with one another [49,50]. Further experiments varying ‘nearest neighbor’ distances would be necessary to shed more light on these questions.

Our observations may have important implications for TE and regenerative applications of hMSC. Many TE approaches require scaffolds to be seeded at high densities to mimic the cellularity of native tissues and create sufficient cell-secreted ECM to form a functional graft. Indeed, many efforts rely on densities far higher than those in our HD cultures [[5], [6], [7]]. Nevertheless, our observations suggest that in HD cultures, the matrix-driven differentiation effect designed into many scaffolds may be countered by a density effect that pushes hMSC towards quiescence. TE strategies are often combined with chemical induction, which may promote differentiation despite a push toward quiescence. However, our observations may provide insight into why direct comparisons between HD cultures of MSC and chondrocytes often show that chondrocytes have better tissue-forming ability, even with identical chemical induction [51]. Our observations may also have important implications for studying quiescence and artificially reproducing SC niches. SC are more potently regenerative if they are quiescent prior to activation [15,52]. However, SC removed from their niche and cultured *in vitro*, lose their quiescent phenotype. By providing appropriate biological and physical stimuli to SC in *ex vivo* native-like niches, it may be possible to create sufficient numbers of therapeutic cells and maintain them in their quiescent, most regenerative state. Indeed, this concept has recently been explored to engineer artificial satellite SC niches that combine defined mechanical properties and ECM compositions with soluble cocktails to enable the maintenance of quiescence [53].

## Declarations of interest

None.

## Author contributions

SAF, APS and EG designed research; SAF, PAF, AJS and TTLY performed research; SL and HWA contributed reagents/analytical tools; SAF, PAF, APS and EG analyzed data; and SAF and EG wrote the paper.
