# Supplementary material for: Neighboring cells override 3D hydrogel matrix cues to drive human MSC quiescence
Source: Biomaterials. 2018 Sep;176:13–23. doi: 10.1016/j.biomaterials.2018.05.032 (PMC6011386; doi:10.1016/j.biomaterials.2018.05.032)
Supplement: Multimedia component 1 [file mmc1.docx]

Supplementary Materials:

**Supplementary Tables**

**Table S1**: Primers used for analyzing gene expression of hMSC encapsulated within S-HA-PEGDA hydrogels.

| Gene | F/R | Sequence | Tm (°C) | Amplicon size | Primer Conc.  (nM) |
| --- | --- | --- | --- | --- | --- |
| *Reference genes* | | | | | |
| *RPL13A* | F | GCC CTA CGA CAA GAA AAA GCG | 60.14 | 117 | 150 |
|  | R | TAC TTC CAG CCA ACC TCG TGA | 61.10 |  |  |
| *EEF1A1* | F | GCT GAG CGT GAA CGT GGT AT | 60.74 | 89 | 150 |
|  | R | CCT GGG GCA TCA ATG ATA GTC A | 59.89 |  |  |
| *Adipogenic genes* | | | | | |
| *PPARγ* | F | TACTGTCGGTTTCAGAAATGCC | 58.93 | 141 | 150 |
|  | R | GTCAGCGGACTCTGGATTCAG | 60.47 |  |  |
| *C/EBPα* | F | GGTGCGTCTAAGATGAGGGG | 59.89 | 141 | 300 |
|  | R | GCATTGGAGCGGTGAGTTTG | 60.11 |  |  |
| *Osteogenic genes* | | | | | |
| *RUNX2* | F | TCAACGATCTGAGATTTGTGGG | 58.40 | 81 | 200 |
|  | R | GGGGAGGATTTGTGAAGACGG | 60.68 |  |  |
| *BGLAP* | F | ATGAGAGCCCTCACACTCCT | 59.96 | 117 | 150 |
|  | R | CTTGGACACAAAGGCTGCAC | 59.97 |  |  |
| *Chondrogenic genes* | | | | | |
| *COL2A1* | F | CCAGATGACCTTCCTACGCC | 59.89 | 186 | 300 |
|  | R | TTCAGGGCAGTGTACGTGAAC | 60.54 |  |  |
| *SOX9* | F | AGCGAACGCACATCAAGAC | 58.85 | 85 | 150 |
|  | R | CTGTAGGCGATCTGTTGGGG | 60.18 |  |  |

Genes: ribosomal protein L13a (*RPL13A*); eukaryotic translation elongation factor 1 alpha 1 (*EEF1A1*); peroxisome proliferator-activated receptor gamma (*PPARγ*); CCAAT/enhancer-binding protein alpha (*C/EBPα*); runt related transcription factor 2 (*RUNX2*); bone gamma-carboxyglutamate protein (*BGLAP*); collagen type II alpha 1 (*COL2A1*); sex determining region Y (SRY)-box 9 (*SOX9*). Primers were designed with Primerbank and Primer Blast and obtained from Integrated DNA Technologies.

**Table S2**: Antibodies used in immunostaining of hMSC encapsulated within S-HA-PEGDA hydrogels.

| Antibody | Dilution | Product code, Supplier |
| --- | --- | --- |
| *Primary antibodies* | | |
| Rabbit monoclonal to Ki67 | 1:100 | ab16667, Abcam |
| Rabbit monoclonal to lumican | 1:50 | ab16834, Abcam |
| Rabbit polyclonal to collagen type II | 1:200 | ab34712, Abcam |
| Rabbit polyclonal to versican | 1:50 | ab19345, Abcam |
| Rat monoclonal to tubulin | 1:400 | ab6161, Abcam |
| *Isotype controls* | | |
| Rabbit IgG monoclonal [EPR25A] | 1:50 | ab172730, Abcam |
| Rabbit IgG polyclonal | 1:200 | ab27478, Abcam |
| Rat IgG2a, κ monoclonal [RTK2758] | 1:400 | ab18450, Abcam |
| *Secondary antibodies* | | |
| Goat polyclonal anti-rabbit biotin | 1:300 | ab6720, Abcam |
| streptavidin, AlexaFluor^®^ 488 conjugate | 1:500 | S11223, Molecular Probes |
| Alexa Fluor^®^ 568 goat anti-rat | 1:300 | ab175476, Abcam |

**Supplementary Figures**

**
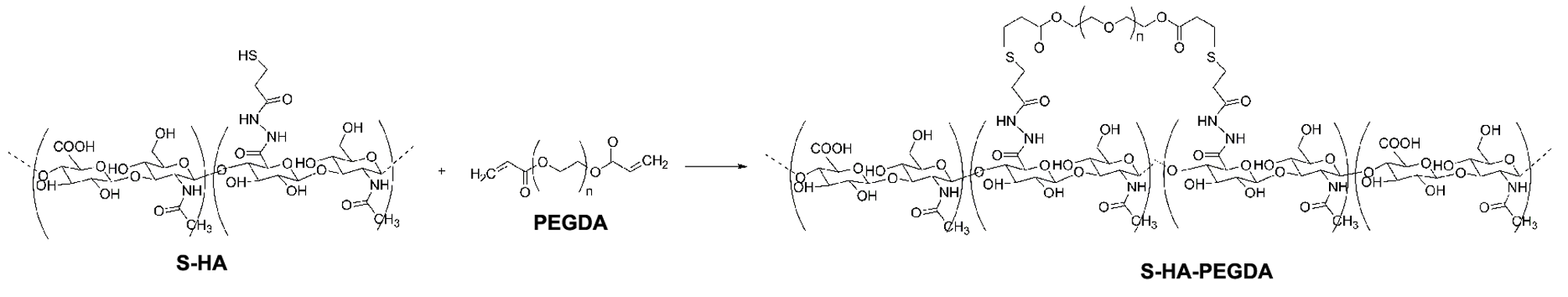
**

**Fig. S1**: Chemical reaction scheme used to form hydrogels. Thiol-modified hyaluronic acid (S-HA) reacts with poly(ethylene glycol) diacrylate (PEGDA) to form hydrogels via a Michael addition. Hydrogels were formed with either a 1:0.375 or 1:0.75 weight ratio of S-HA to PEGDA. This ensured that the concentration of biologically active HA was always constant, but allowed us to alter the swelling, mechanical, and degradative properties of the hydrogels.


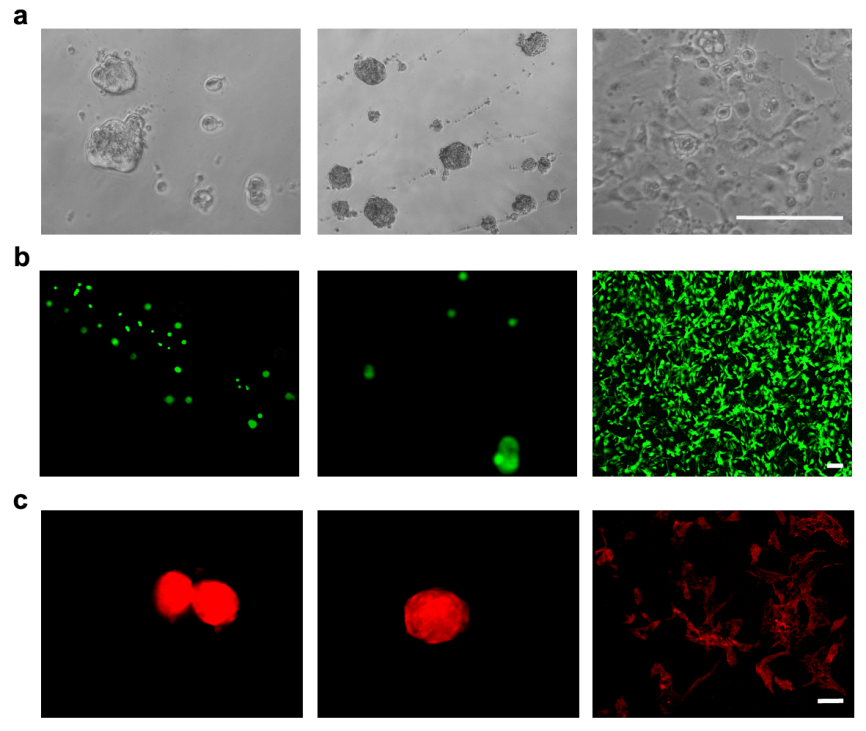


**Fig. S2**: Representative micrographs of hMSC cultured for 1 day on the surface of 1:0.75 S-HA-PEGDA hydrogels (left), S-HA-PEGDA hydrogels with 100 μg/mL fibronectin from bovine plasma (middle), or S-HA-PEGDA modified with 1% thiol-modified gelatin at a density of 3.5x10^4^ cells/cm^2^. Micrographs in **a**. show differences in cell attachment by phase contrast microscopy on different surfaces before washing, in **b**. show hMSC stained with LIVE/DEAD^®^ after media change, and in **c.** show hMSC stained with phalloidin-TRITC (red). Images show limited attachment and cell spreading on S-HA-PEGDA hydrogels even after adding fibronectin. Fibronectin was not chemically bound to the hydrogel. Therefore, as free sulphydryl groups in fibronectin are not available [[1](#_ENREF_1)] to interact with acrylate or other reactive groups in the hydrogel, cells do not attach. S-HA-PEGDA hydrogels modified with 1% thiol-modified gelatin, however, support cell attachment and spreading. Scale bar **a**. and **b**. = 100 µm. Scale bar **c**. = 200 µm.

**
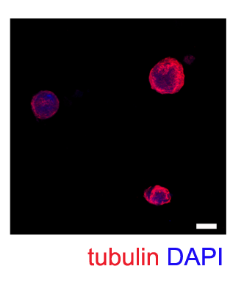
**

**Fig. S3:** Representative micrograph from a HD culture of hMSC encapsulated within a 1:0.75 S-HA-PEGDA hydrogel after 4 h in culture. All cells adopted round morphologies. Scale bar = 10 µm.


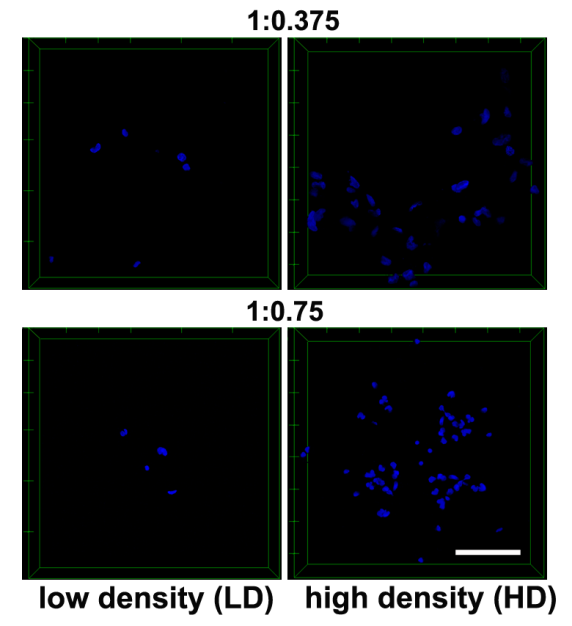


**Fig. S4:** Representative micrographs of LD and HD cultures of hMSC in 1:0.375 and 1:0.75 S-HA-PEGDA hydrogels after 72 h in control media (no HPG). Fluorescence tagging with the Alexa Fluor 488 azide was highly specific. Scale bar = 100 µm.

**
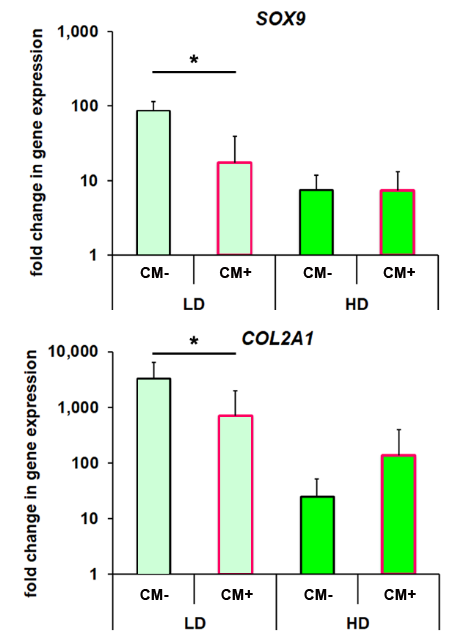
**

**Fig. S5:** Gene expression analyses for markers of chondrogenesis (*SOX9* and *COL2A1*) in LD and HD cultures of hMSC 72 h after encapsulation in 1:0.75 S-HA-PEGDA hydrogels. Expression is normalized to undifferentiated hMSC for controls cultured with basal medium (CM-) or treated with conditioned medium (CM+, outlined pink) (*n* ≥ 6, **P* < 0.05). Plots show mean + SD. A two-tailed Mann-Whitney test was used to detect statistical significance.


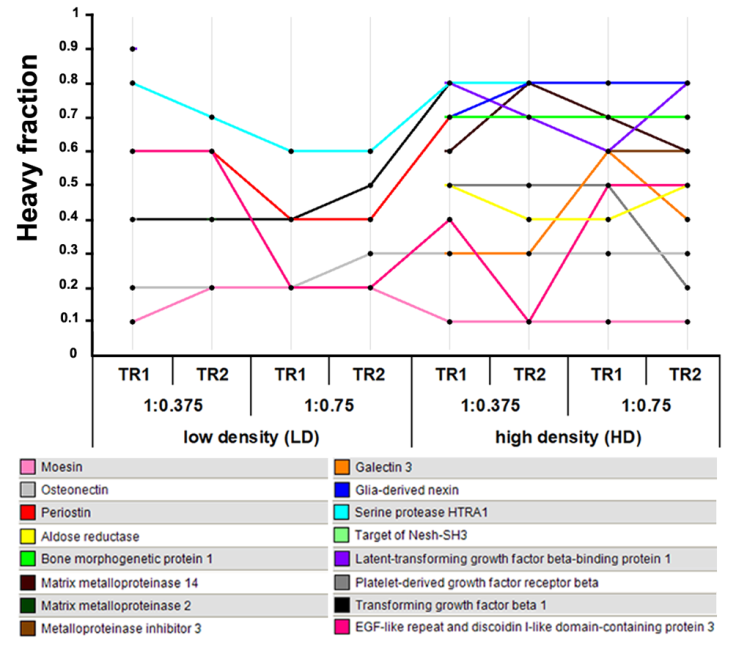


**Fig. S6:** Profile plots showing the heavy fraction of a selection of labelled functional/regulatory proteins produced by LD and HD cultures of hMSC in 1:0.375 and 1:0.75 S-HA-PEGDA hydrogels (two technical replicates, TR) after 72 h in culture. Proteins known to play functional or regulatory roles in adult stem cells niches showed high levels of incorporation of the heavy label in HD cultures. In LD cultures, levels of incorporation were lower or we could not calculate a ratio.


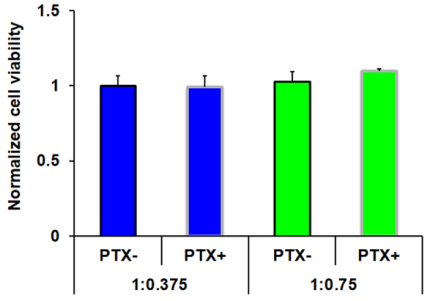


**Fig. S7:** Viability of hMSC in HD cultures encapsulated in 1:0.375 and 1:0.75 S-HA-PEGDA hydrogels (*n* = 3) for 72 h and treated with 50 nM paclitaxel *(*PTX+) or the vehicle control (PTX-). Values are normalized to vehicle controls and plots show the mean + SD. A two-tailed Mann-Whitney test was used to detect statistical significance.

**
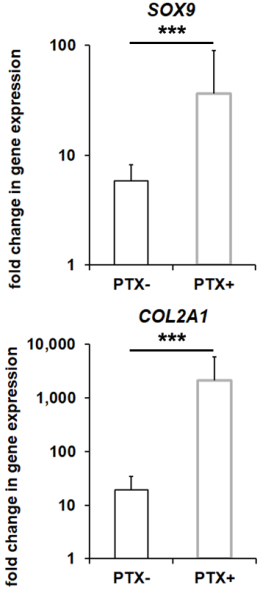
**

**Fig. S8:** Gene expression analyses for markers of chondrogenesis (*SOX9* and *COL2A1*) in HD hMSC cultures treated for 72 h with paclitaxel (PTX+, outlined grey) or the vehicle control (PTX-) and normalized to undifferentiated hMSC controls (*n* ≥ 16, ****P* < 0.001). Plots shown mean + SD and a two-tailed Mann-Whitney test was used to detect statistical significance.

**Data file S1**: Analyzed proteomics data set. Data are provided as a detailed excel file.

**References**

[1] D.F. Mosher, R.B. Johnson. In vitro formation of disulfide-bonded fibronectin multimers. J. Biol. Chem. 258 (1983) 6595-6601.
